# Supplementary material for: Effects of treatment with enrofloxacin or tulathromycin on fecal microbiota composition and genetic function of dairy calves
Source: PLoS One. 2019 Dec 11;14(12):e0219635. doi: 10.1371/journal.pone.0219635 (PMC6905572; doi:10.1371/journal.pone.0219635)
Supplement: S1 Table — More information is available at MG-RAST in project 20043 (https://www.mg-rast.org/linkin.cgi?project=mgp20043). (PDF) [file pone.0219635.s001.pdf]

Table S1: Sequencing data by sample. More information is available at MG-RAST in project 20043 (https://www.mg-rast.org/linkin.cgi?project=mgp20043).

MG-RAST ID	Block	Study group	Sampling Day	Total sequences (bp)	Sequences read (bp)
4718754.3	1	CON	0	412,294,106	1,642,606
4736314.3	1	CON	4	412,260,472	1,642,472
4718752.3	1	CON	14	329,518,322	1,312,822
4736311.3	1	CON	56	400,398,212	1,595,212
4736307.3	1	CON	112	425,552,428	1,695,428
4718760.3	1	CON	0	211,864,080	844,080
4736308.3	1	CON	4	243,746,100	971,100
4718775.3	1	CON	14	267,475,640	1,065,640
4736312.3	1	CON	56	319,436,656	1,272,656
4736316.3	1	CON	112	306,153,234	1,219,734
4718771.3	2	CON	0	325,162,468	1,295,468
4736970.3	2	CON	4	363,791,368	1,449,368
4718766.3	2	CON	14	503,211,828	2,004,828
4736964.3	2	CON	56	456,894,798	1,820,298
4736967.3	2	CON	112	480,356,270	1,913,770
4718781.3	2	CON	0	384,765,430	1,532,930
4736977.3	2	CON	4	333,027,804	1,326,804
4718774.3	2	CON	14	206,417,380	822,380
4736974.3	2	CON	56	209,935,396	836,396
4736979.3	2	CON	112	350,511,460	1,396,460
4718773.3	3	CON	0	257,345,280	1,025,280
4748303.3	3	CON	4	311,650,143	1,154,973
4718757.3	3	CON	14	516,964,118	2,059,618
4737005.3	3	CON	56	636,123,356	2,534,356
4737004.3	3	CON	112	285,818,218	1,138,718
4718782.3	3	CON	0	404,583,386	1,611,886
4737011.3	3	CON	4	322,199,162	1,283,662
4718772.3	3	CON	14	252,813,726	1,007,226
4736987.3	3	CON	56	521,550,892	2,077,892
4737012.3	3	CON	112	444,087,272	1,769,272
4737463.3	4	CON	0	450,066,092	1,793,092
4737432.3	4	CON	4	417,241,818	1,662,318
4737448.3	4	CON	14	260,557,578	1,038,078
4737441.3	4	CON	56	129,241,908	514,908
4737455.3	4	CON	112	171,589,122	683,622
4737460.3	4	CON	0	247,513,610	986,110
4737464.3	4	CON	4	179,067,918	713,418
4737450.3	4	CON	14	302,280,304	1,204,304
4737445.3	4	CON	56	289,224,790	1,152,290
4737431.3	4	CON	112	219,494,982	874,482
4737451.3	5	CON	0	292,244,822	1,164,322
4737452.3	5	CON	4	278,225,970	1,108,470
4737447.3	5	CON	14	224,375,426	893,926
4737430.3	5	CON	56	179,804,352	716,352
4737461.3	5	CON	112	358,654,904	1,428,904

MG-RAST ID	Block	Study group	Sampling Day	Total sequences (bp)	Sequences read (bp)
4739043.3	5	CON	0	320,740,350	1,277,850
4739033.3	5	CON	4	292,225,746	1,164,246
4739038.3	5	CON	14	292,769,914	1,166,414
4739031.3	5	CON	56	346,749,974	1,381,474
4739036.3	5	CON	112	158,967,336	633,336
4739039.3	6	CON	0	348,846,326	1,389,826
4739042.3	6	CON	4	300,183,450	1,195,950
4739037.3	6	CON	14	262,246,808	1,044,808
4739047.3	6	CON	56	213,012,154	848,654
4739044.3	6	CON	112	192,328,750	766,250
4748339.3	6	CON	0	201,979,198	804,698
4748337.3	6	CON	4	235,965,100	940,100
4748330.3	6	CON	14	282,478,914	1,125,414
4748329.3	6	CON	56	276,723,986	1,102,486
4748334.3	6	CON	112	184,571,344	735,344
4718767.3	1	ENR	0	389,753,804	1,552,804
4736315.3	1	ENR	4	459,958,002	1,832,502
4718787.3	1	ENR	14	357,868,270	1,425,770
4736306.3	1	ENR	56	340,111,526	1,355,026
4736313.3	1	ENR	112	320,645,974	1,277,474
4718778.3	1	ENR	0	378,351,376	1,507,376
4736448.3	1	ENR	4	476,748,396	1,899,396
4718763.3	1	ENR	14	368,960,964	1,469,964
4737466.3	1	ENR	56	508,219,278	2,024,778
4736965.3	1	ENR	112	568,551,144	2,265,144
4718777.3	2	ENR	0	527,603,506	2,102,006
4736975.3	2	ENR	4	434,768,646	1,732,146
4718765.3	2	ENR	14	552,944,466	2,202,966
4736981.3	2	ENR	56	420,978,204	1,677,204
4736980.3	2	ENR	112	380,038,096	1,514,096
4718764.3	2	ENR	0	202,356,200	806,200
4736978.3	2	ENR	4	558,617,568	2,225,568
4718753.3	2	ENR	14	509,188,640	2,028,640
4737010.3	2	ENR	56	530,937,790	2,115,290
4736997.3	2	ENR	112	285,326,760	1,136,760
4718758.3	3	ENR	0	446,905,500	1,780,500
4737001.3	3	ENR	4	251,662,640	1,002,640
4718761.3	3	ENR	14	183,951,876	732,876
4737007.3	3	ENR	56	148,812,880	592,880
4736994.3	3	ENR	112	41,750,336	166,336
4718759.3	1	ENR	0	445,882,926	1,776,426
4737003.3	1	ENR	4	326,568,570	1,301,070
4718784.3	1	ENR	14	31,273,094	124,594
4736996.3	1	ENR	56	276,240,058	1,100,558
4736990.3	1	ENR	112	150,856,020	601,020

MG-RAST ID	Block	Study group	Sampling Day	Total sequences (bp)	Sequences read (bp)
4736989.3	2	ENR	0	200,600,204	799,204
4736992.3	2	ENR	4	476,617,374	1,898,874
4736993.3	2	ENR	14	350,210,260	1,395,260
4736998.3	2	ENR	56	236,354,652	941,652
4736999.3	2	ENR	112	421,909,916	1,680,916
4737433.3	4	ENR	0	321,270,462	1,279,962
4737436.3	4	ENR	4	329,014,816	1,310,816
4737458.3	4	ENR	14	355,583,166	1,416,666
4737449.3	4	ENR	56	260,582,176	1,038,176
4737467.3	4	ENR	112	299,448,522	1,193,022
4737429.3	5	ENR	0	329,744,724	1,313,724
4737438.3	5	ENR	4	59,715,912	237,912
4737437.3	5	ENR	14	197,955,166	788,666
4737435.3	5	ENR	56	122,998,032	490,032
4737459.3	5	ENR	112	82,031,820	326,820
4738993.3	5	ENR	0	194,093,280	773,280
4739041.3	5	ENR	14	297,736,200	1,186,200
4738994.3	5	ENR	56	260,034,996	1,035,996
4738996.3	5	ENR	112	289,287,540	1,152,540
4739049.3	6	ENR	0	206,491,174	822,674
4744057.3	6	ENR	4	269,349,104	1,073,104
4748335.3	6	ENR	14	181,913,756	724,756
4748336.3	6	ENR	56	101,538,536	404,536
4748340.3	6	ENR	112	290,108,310	1,155,810
4748342.3	6	ENR	0	383,025,498	1,525,998
4748325.3	6	ENR	4	360,088,114	1,434,614
4748338.3	6	ENR	14	183,658,206	731,706
4748333.3	6	ENR	56	233,778,388	931,388
4748328.3	6	ENR	112	181,691,872	723,872
4718756.3	1	TUL	0	343,505,548	1,368,548
4736317.3	1	TUL	4	304,028,268	1,211,268
4718768.3	1	TUL	14	306,821,898	1,222,398
4736310.3	1	TUL	56	336,623,630	1,341,130
4736309.3	1	TUL	112	234,000,272	932,272
4718786.3	1	TUL	0	200,737,752	799,752
4736449.3	1	TUL	4	263,393,376	1,049,376
4718755.3	1	TUL	14	289,268,966	1,152,466
4736447.3	1	TUL	56	273,331,470	1,088,970
4736450.3	1	TUL	112	375,827,822	1,497,322
4718769.3	2	TUL	0	676,843,588	2,696,588
4736966.3	2	TUL	4	442,124,452	1,761,452
4718779.3	2	TUL	14	384,077,188	1,530,188
4736969.3	2	TUL	56	390,167,954	1,554,454
4736968.3	2	TUL	112	260,423,042	1,037,542

MG-RAST ID	Block	Study group	Sampling Day	Total sequences (bp)	Sequences read (bp)
4718783.3	2	TUL	0	467,377,060	1,862,060
4736982.3	2	TUL	4	519,494,198	2,069,698
4718780.3	2	TUL	14	514,384,842	2,049,342
4736973.3	2	TUL	56	601,748,906	2,397,406
4736976.3	2	TUL	112	348,261,998	1,387,498
4718762.3	3	TUL	0	531,542,198	2,117,698
4736991.3	3	TUL	4	627,802,706	2,501,206
4718785.3	3	TUL	14	506,421,616	2,017,616
4736995.3	3	TUL	56	614,615,166	2,448,666
4737002.3	3	TUL	112	380,107,874	1,514,374
4718776.3	3	TUL	0	407,544,182	1,623,682
4737000.3	3	TUL	4	428,804,886	1,708,386
4718770.3	3	TUL	14	456,842,590	1,820,090
4737009.3	3	TUL	56	566,968,338	2,258,838
4736984.3	3	TUL	112	435,824,854	1,736,354
4736988.3	4	TUL	0	234,462,614	934,114
4736985.3	4	TUL	4	397,892,228	1,585,228
4736986.3	4	TUL	14	509,178,600	2,028,600
4737006.3	4	TUL	56	342,213,902	1,363,402
4737008.3	4	TUL	112	187,559,750	747,250
4737446.3	4	TUL	0	249,707,350	994,850
4737428.3	4	TUL	4	256,226,824	1,020,824
4737462.3	4	TUL	14	332,893,268	1,326,268
4737444.3	4	TUL	56	309,580,890	1,233,390
4737440.3	4	TUL	112	213,076,410	848,910
4737465.3	5	TUL	0	164,318,656	654,656
4737442.3	5	TUL	4	199,653,432	795,432
4737453.3	5	TUL	14	181,951,908	724,908
4737443.3	5	TUL	56	241,137,206	960,706
4737439.3	5	TUL	112	288,980,818	1,151,318
4737468.3	5	TUL	0	297,134,302	1,183,802
4737456.3	5	TUL	4	246,713,422	982,922
4737434.3	5	TUL	14	286,269,516	1,140,516
4737454.3	5	TUL	56	202,924,464	808,464
4737457.3	5	TUL	112	212,561,358	846,858
4739048.3	6	TUL	0	30,653,626	122,126
4739035.3	6	TUL	4	230,322,118	917,618
4739040.3	6	TUL	14	326,317,068	1,300,068
4739028.3	6	TUL	56	435,625,058	1,735,558
4739045.3	6	TUL	112	261,531,960	1,041,960
4748331.3	6	TUL	0	286,624,932	1,141,932
4748327.3	6	TUL	4	311,182,772	1,239,772
4748341.3	6	TUL	14	176,779,802	704,302
4748326.3	6	TUL	56	149,518,692	595,692
4748332.3	6	TUL	112	187,480,936	746,936
