# Supplementary figures and images for: Effects of treatment with enrofloxacin or tulathromycin on fecal microbiota composition and genetic function of dairy calves
Source: PLoS One. 2019 Dec 11;14(12):e0219635. doi: 10.1371/journal.pone.0219635 (PMC6905572; doi:10.1371/journal.pone.0219635)

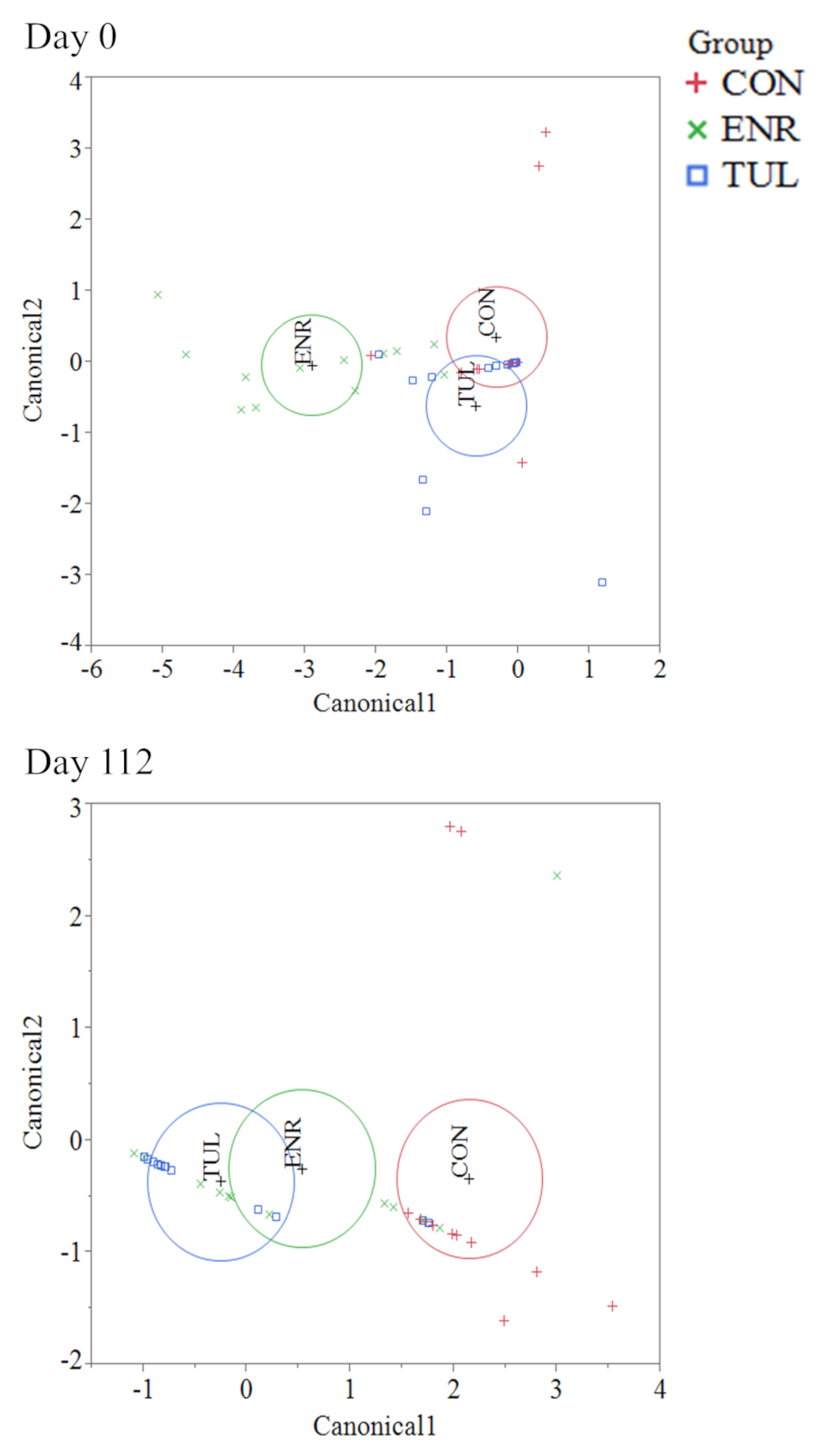

Supplement: S1 Fig — Changes for each study group on Day 0 and Day 112 after enrollment (P-value < 0.05). An ellipse indicates the 95% confidence region to contain the true mean of the variable (group). CON = control, ENR = enrofloxacin, TUL = tulathromycin. (TIF) [file pone.0219635.s002.tif]

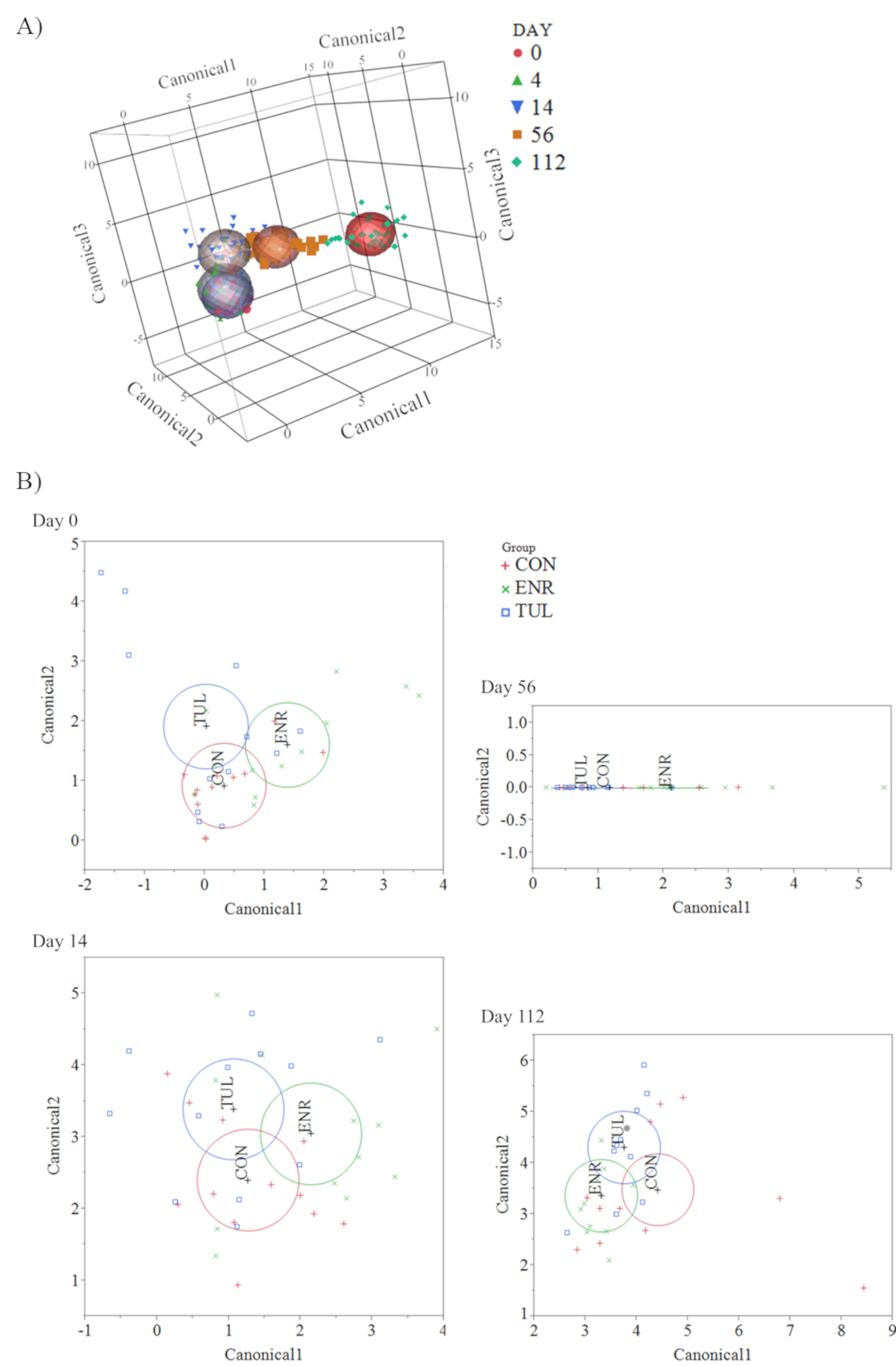

Supplement: S2 Fig — A) Changes in genera composition over time (P-value < 0.05). B) Changes for each study group by days after enrollment. An ellipse indicates the 95% confidence region to contain the true mean of the variable (day or group). CON = control, ENR = enrofloxacin, TUL = tulathromycin. (TIF) [file pone.0219635.s003.tif]

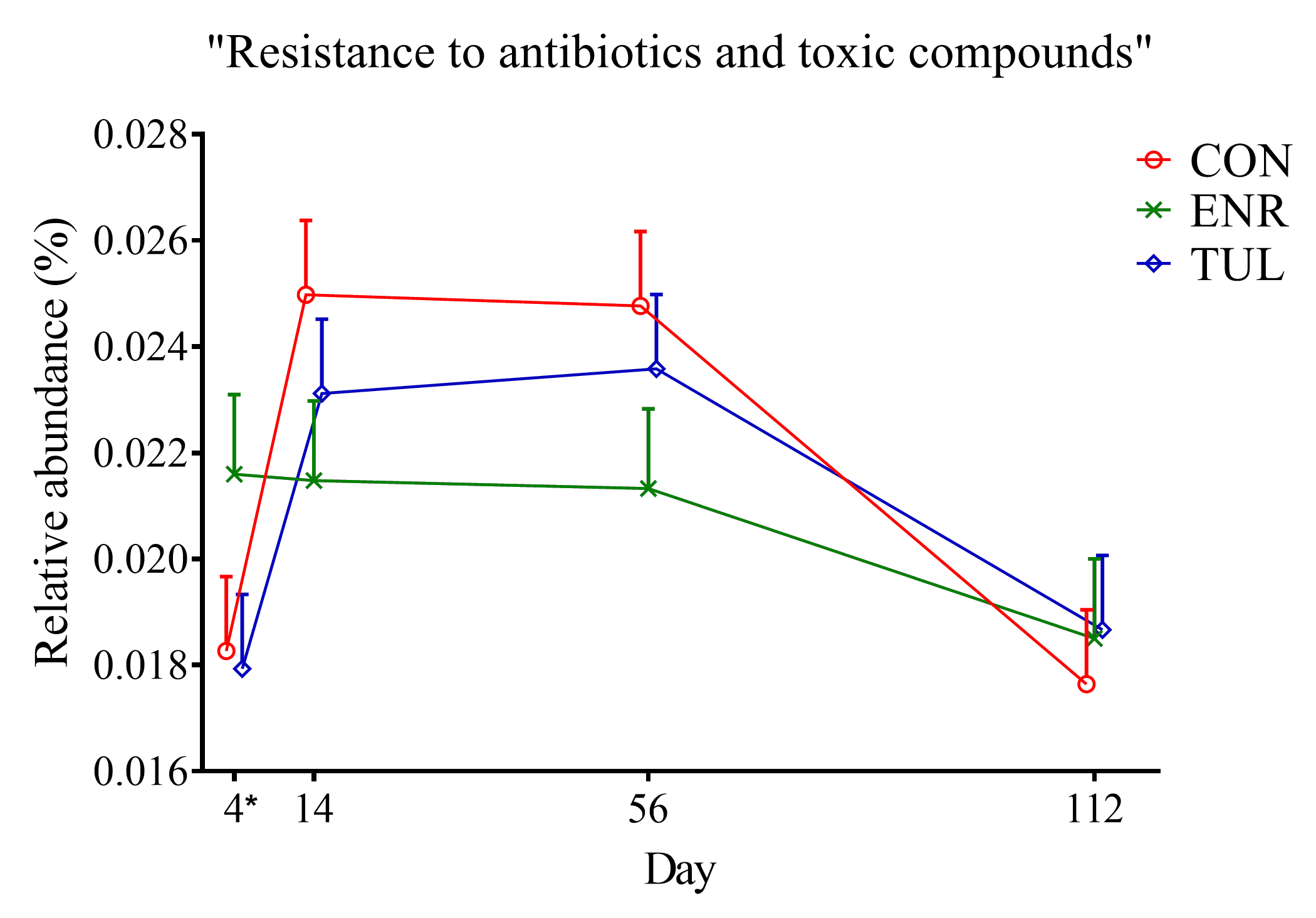

Supplement: S3 Fig — The independent variables study group (CON = control, ENR = enrofloxacin, TUL = tulathromycin), sample day (4, 14, 56, and 112) and interactions were included as fixed effects in all models. Day 0 was included as a covariate in the model. The effects block and individual animals nested within block were controlled in the models as random effects. Asterisks indicate significant differences (P-value ≤ 0.05) between day and study group. Error bars represent the standard error of the least square mean. (TIF) [file pone.0219635.s004.tif]
